# Flies do the locomotion

**DOI:** 10.7554/eLife.10317

**Published:** 2015-08-28

**Authors:** Jennifer K Lovick, Jaison J Omoto, Volker Hartenstein

**Affiliations:** Department of Molecular, Cell, and Developmental Biology, University of California, Los Angeles, Los Angeles, United States; Department of Molecular, Cell, and Developmental Biology, University of California, Los Angeles, Los Angeles, United States; Department of Molecular, Cell, and Developmental Biology, University of California, Los Angeles, Los Angeles, United Statesvolkerh@mcdb.ucla.edu

**Keywords:** neuroblasts, lineages, flight, walking, insects, *D. melanogaster*

## Abstract

Genetic techniques have shed new light on the organization of the neurons in the ventral nervous system of the fruit fly.

**Related research article** Harris RM, Pfeiffer BD, Rubin GM, Truman JW. 2015. Neuron hemilineages provide the functional ground plan for the *Drosophila* ventral nervous system. *eLife* **4**:e04493. doi: 10.7554/eLife.04493

**Image** A decapitated fly moves in response to the stimulation of one of its hemilineages

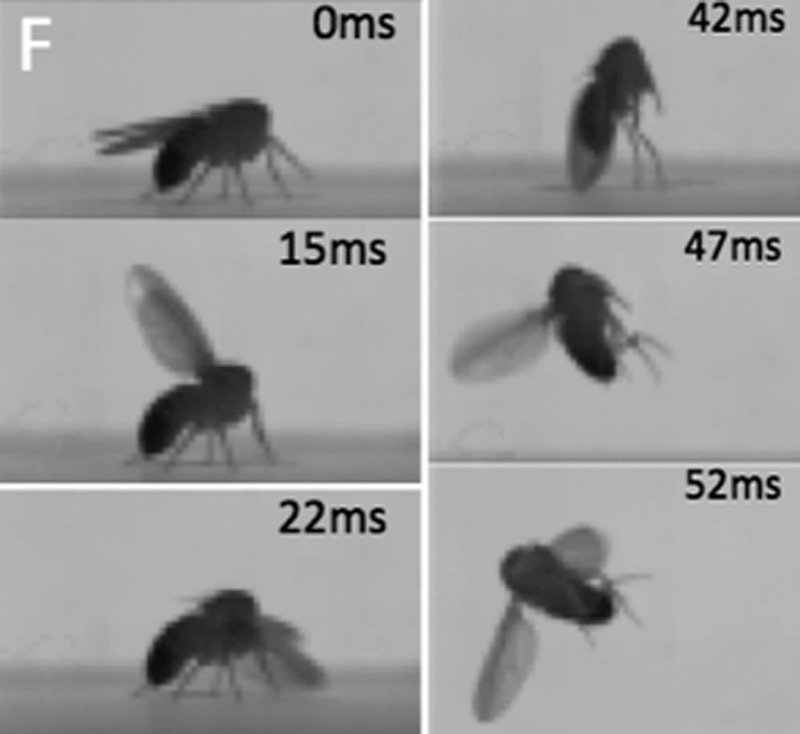

Neurons can be divided into classes based on their structure, and many animal movements (locomotive behaviors) are controlled by circuits that contain more than one class of neuron. Some of these movements are relatively simple, such as the twitch of a limb, whereas others are more complex, such as walking and flying (Garcia-Campmany et al., 2010; Grillner and El Manira, 2015). Studies of both invertebrate and vertebrate neuronal circuits have provided an increasing amount of evidence that neurons in the same class develop from the same progenitor cell and/or progenitor cells. Now, in eLife, Jim Truman and colleagues at the Janelia Research Campus – including Robin Harris as first author – report how different developmentally-related classes of neurons in the ventral nervous system of the fruit fly *Drosophila* work to control movement (Harris et al., 2015).

In the fruit fly and other insects, the ventral nervous system is the equivalent of the vertebrate spinal cord, and develops from progenitor cells called neuroblasts. Neuroblasts divide to produce a new neuroblast and a cell called a ganglion mother cell (Figure 1). The ganglion mother cell then divides to produce two daughter cells that develop into neurons. Each neuroblast undergoes multiple divisions to generate its own unique set of neural progeny called a lineage. There are approximately thirty paired neuroblasts in each segment of the *Drosophila* ventral nervous system, and hence thirty different lineages of neurons.

**Figure 1.**
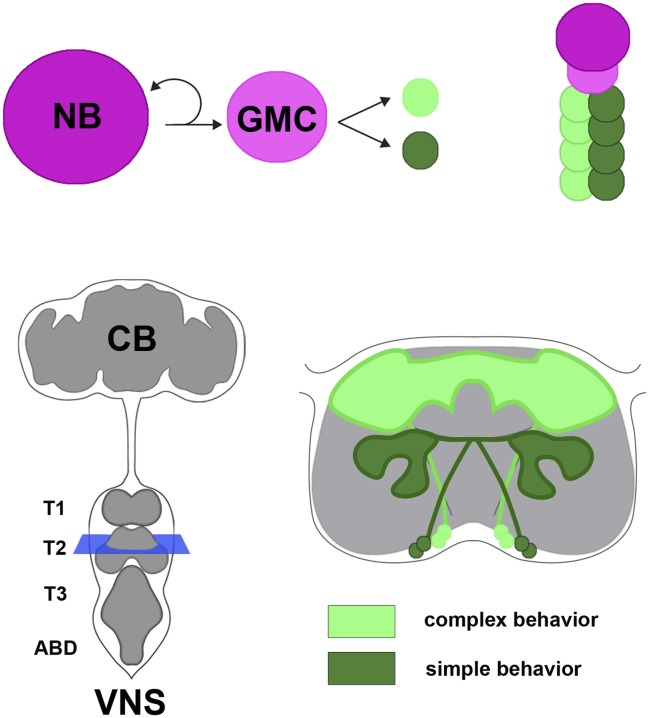
Neural hemilineages and the ventral nervous system of the fruit fly. A neuroblast (NB) divides asymmetrically to produce a new neuroblast and a ganglion mother cell (GMC; top left). The GMC then divides symmetrically to produce two daughter neurons that belong to different hemilineages (represented by dark green and light green circles; top right). The central nervous system consists of the central brain (CB) and the ventral nervous system (VNS; bottom left). Harris et al. looked at the anatomy and function of hemilineages in the VNS, using new genetic techniques to label individual hemilineages in order to study their branching pattern. They found that hemilineages with relatively simple branching patterns (dark green; bottom right) control simple behaviors, whereas hemilineages with more intricate branching patterns (light green) tend to control complex behaviors. The schematic shown on the bottom right of the figure corresponds to the blue square in the bottom left.

Previous work from the Truman lab had shown that the neurons which develop from each ganglion mother cell fall into two hemilineages; neurons that exhibit high activity of the Notch gene belong to the A hemilineage, whereas neurons that exhibit low activity belong to the B hemilineage (Truman et al., 2010). Thus, a given lineage is made of A and B hemilineages. Neurons of a hemilineage tend to cluster together so that their axons form a coherent bundle that projects to a target region within the ventral nervous system.

To investigate the role of hemilineages in the circuits that control locomotion, Harris et al. had to develop a suite of genetic tools that allowed them to permanently label a given hemilineage without labeling other cells in the ventral nervous system. Almost every hemilineage of the ventral nervous system could be targeted by using these tools in conjunction with the large existing collections of genetically engineered *Drosophila* lines (Pfeiffer et al., 2008; Jenett et al., 2012). By labeling hemilineages in this way, Harris et al. were able to build an anatomical ‘roadmap’ of the circuitry that controls how the fly moves.

Modifying the neurons in a specific hemilineage so that they expressed a heat-sensitive channel called dTRPA1 allowed that hemilineage to be activated by increasing the temperature. Using this technique, Harris et al. could investigate the behaviors controlled by individual hemilineages. They found that activating a specific hemilineage typically caused the fly to make a particular movement. For example, one hemilineage controls a particular type of leg stretch, and another allows the fly to take off for flight. To demonstrate that these behavioral responses were not due to unintentionally activated brain neurons, Harris et al. used decapitated flies. These decapitated flies will stand for some time with a robust posture and even undergo bouts of simple grooming behaviors.

As might be expected, the behaviors produced by activating specific hemilineages were typically related to the branching patterns of the neurons (Figure 1). For example, hemilineages that projected to the so-called leg neuropil elicited a leg-related behavior; likewise for other regions in the ventral nervous system, such as those that correspond to the wing. Examining the structure of the cells and the behaviors they control revealed a number of organizational principles. For one, Harris et al. observed that “homogenous” hemilineages contained fewer neuron types and simple branching patterns, and so proposed that these represent parallel collections of neurons that transmit specific types of information in a more linear fashion. In contrast, hemilineages that were “heterogeneous” had more complex branching patterns and are thought to have diverse functions, possibly integrating information from multiple sources. Most importantly, simple movements like leg stretches and twitches were typically controlled by the more ventrally-located simple hemilineages. More complex behaviors (such as walking, wing waving, or the sequential movements needed for flight take-off) were controlled by the typically more complex hemilineages located dorsally towards the fly's back.

The nervous systems of other insect species are almost certainly organized into a hierarchy based on an arrangement of hemilineages (Thomas et al., 1984). The analysis of Harris et al. paves the way towards uncovering the hemilineage plan in more evolutionarily basal groups of insects, notably orthoptera (which includes grasshoppers and locusts) and other insects that do not go through a pupal stage. Because of their large and experimentally accessible neurons, these insects have traditionally been used to dissect the roles of the individual components of the circuits that regulate movement (Burrows, 1992; Büschges et al., 2008).

Approaching the analysis of neuronal activity and behavior on the basis of the hemilineage roadmap also promises to provide significant steps forward in our understanding of the circuitry that controls movement in insects and other animals. In vertebrate nervous systems, the patterns of activity that control a particular behavior emerge from the combined activity of large networks of neurons. Harris et al. demonstrate that developmentally-related neuronal classes, rather than individual neurons, form the basic units of locomotor circuitry, a concept that likely directly applies to vertebrates.
